# Supplementary material for: Chemometrics-Assisted Identification of Anti-Inflammatory Compounds from the Green Alga Klebsormidium flaccidum var. zivo
Source: Molecules. 2020 Feb 26;25(5):1048. doi: 10.3390/molecules25051048 (PMC7179104; doi:10.3390/molecules25051048)
Supplement: Supplementary file 1 [file molecules-25-01048-s001.pdf]

molecules

Supplementary Material

Chemometrics-Assisted Identification of Anti-inflammatory Compounds from the Green Alga *Klebsormidium flaccidum* var. *zivo*

Shi Qiu [1], Shabana I. Khan [1,2], Mei Wang [1], Jianping Zhao [1], Siyu Ren [1], Ikhlas A. Khan [1,2], Amy Steffek [3], William P. Pfund [3] and Xing-Cong Li [1,2,*]

[1] National Center for Natural Product Research, Research Institute of Pharmaceutical Sciences, School of Pharmacy, The University of Mississippi, University, Mississippi 38677, United States; davidhugh@msn.cn (S.Q.), skhan@olemiss.edu (S.I.K.), meiwang@olemiss.edu (M.W.), jianping@olemiss.edu (J.Z.), siyuren07@163.com (S.R.), ikhan@olemiss.edu (I.A.K.)

[2] Department of Biomolecular Sciences, School of Pharmacy, The University of Mississippi, University, MS 38677, United States

[3] ZIVO Biosciences, INC, 2804 Orchard Lake Road, Suite 202, Keego Harbor, MI 48320, United States; asteffek@zivobioscience.com (A.S.), wpfund@zivobioscience.com (W.P.P.)

* Correspondence: xcli7@olemiss.edu; Tel.: +662-915-6742

1. Isolation of anti-inflammatory marker compounds from active column fractions

Fr. 8 (470.0 mg) was absorbed on reverse-phase silica gel (1.0 g) and loaded on a reverse-phase silica gel column (70.0 g). The column was eluted with an isocratic solvent system consisting of acetonitrile and water in a ratio of 55:45 (*v*/*v*) to yield 200 subfractions (A1-A200). Subfractions A77−A85, A103−A109 and A165−A176 were combined to give (10*E*,12*Z*,15*Z*)-9-hydroxyoctadecadienoic acid (**21**) (16.0 mg), (10*E*,12*Z*)-9-hydroxy-octadecadienoic acid (**22**) (39.0 mg), and (9*Z*,12*Z*)-octadecadienamide (**24**) (14.0 mg), respectively. Identification of these compounds was made by comparison of their NMR data with those reported in the literature (Murakami et al. *Lipids* 1992, 27, 776−778; Dabur & Mittal. *Alcohol* 2016, 52, 71−78).

Fr. 10 (200.0 mg) was absorbed on reverse-phase silica gel (0.4 g) and loaded on a reverse-phase silica gel column (50.0 g). The column was eluted with an isocratic solvent system consisting of acetonitrile and water in a ratio of 90:10 (*v*/*v*) to yield 52 subfractions (B1-B52). Subfractions B27−B49 were combined to afford pheophobide a (**26**) (85.0 mg). Identification of this compound was made by comparison of its NMR data with those reported in the literature (Islam et al., *Food Chem. Toxicol.* 2013, 55, 541−548.).

Fr. 12 (190.0 mg) was absorbed on normal-phase silica gel (0.3 g) and loaded on a normal-phase silica gel column (50.0 g). The column was eluted with a gradient solvent system consisting of chloroform and methanol in ratios of 50:1, 20:1 and 10:1 (*v*/*v*) to yield 110 subfractions (C1-C110). Subfractions C77−C101 were combined to afford (2*S*)-1-O-(9*Z*, 12*Z*, 15*Z*-octadecatrinoyl)-2-*O*-(4*Z*, 7*Z*, 10*Z*, 13*Z*-hexadecatetranoyl)-3-*O*-β-D-galactopyranosyl- glycerol (**31**) (60.0 mg). Identification of this compound was made by comparison of its NMR data with those reported in the literature (Banskota et al., *Nat. Prod. Res.* 2012, 27, 1084−1090).

Fr. 19 (190.0 mg) was separated by preparative HPLC using a Gemini 5 μm C_{18} 110A column (10.0 × 250 nm, Phenomenix Co., Ltd.) and a solvent system consisting of 0.1% formic acid in 85% acetonitrile in water at a flow rate of 2.5 mL/min and UV detection at 400 nm, yielding hydro-pheophorbide-lactone a (**42**) (3.8 mg). Identification of this compound was made by comparison of its NMR data with those reported in the literature (Zhao et al., Food Chem. 2014,151, 101−109). Complete assignments of its ^{1}H and ^{13}C signals were made by 2D NMR spectra (COSY, HMQC, HMBC, and ROESY) and are shown in Table S5.

1. GC-MS analysis of non-hydroxylated fatty acids

20 mg of the algal biomass was added to 1 mL 0.4 M KOH/MeOH, sonicated for 1 h, and then extracted with 1 mL of *n*-hexane for 30 min twice. 2 μL of the analyte was injected into instrument for analysis. Gas chromatographic analysis was performed on an Agilent 7890 GC instrument equipped with an Agilent 5975C mass detector and an Agilent 7693 auto-sampler. A silica capillary column (60 m, 0.25 mm ID, 0.2 μm film thickness, Agilent, J&W HP-88) was used with helium as the carrier gas at a flow rate of 1.2 mL/min. The oven was held at 60 °C for 1 min, then programmed at 10 °C/min to 145 °C, 1 °C/min to 190 °C, and 5 °C/min to 220 °C. The injector temperature was 250 °C. The split ratio was set to 50:1. The total ion current chromatogram is shown in Figure S1. Mass spectra were recorded at 70 eV from *m/z* 50 to 550. Fatty acids were identified by direct comparisons of their mass spectral patterns to the mass spectral database (Wiley and NIST) and their retention times with those of 37 known standard FAMEs.

Table 1. Column fractions from ethyl acetate extract and their in vitro anti-inflammatory activities.

Fraction	Eluting System	Weight (mg)	iNOS Inhibition (IC_{50}, μg/mL)	NF-kB Inhibition (IC_{50}, μg/mL)
Fr. 1	$CHCl_3$ = 100%	987.8	NA[a]	NA
Fr. 2	$CHCl_3$ =100%	6207.5	>50[b]	NA
Fr. 3	$CHCl_3$ =100%	2726.9	NA	NA
Fr. 4	$CHCl_3$:MeOH =100:1	298.4	34	NA
Fr. 5	$CHCl_3$:MeOH =100:1	152.2	28	NA
Fr. 6	$CHCl_3$:MeOH =100:1	1241.4	>50	NA
Fr. 7	$CHCl_3$:MeOH =50:1	300.9	23	NA
Fr. 8	$CHCl_3$:MeOH =50:1	524.8	18	NA
Fr. 9	$CHCl_3$:MeOH =50:1	439.8	6.5	NA
Fr. 10	$CHCl_3$:MeOH =20:1	248.3	1.7	32
Fr. 11	$CHCl_3$:MeOH =20:1	396.8	16	NA
Fr. 12	$CHCl_3$:MeOH =20:1	210.7	19	NA
Fr. 13	$CHCl_3$:MeOH =10:1	89.4	16	NA
Fr. 14	$CHCl_3$:MeOH =10:1	108.9	18	NA
Fr. 15	$CHCl_3$:MeOH =10:1	35.3	30	NA
Fr. 16	$CHCl_3$:MeOH =5:1	49.9	39	NA
Fr. 17	$CHCl_3$:MeOH =5:1	89.9	17	NA
Fr. 18	$CHCl_3$:MeOH =5:1	82.6	16	NA
Fr. 19	$CHCl_3$:MeOH =1:1	198.5	25	NA
Fr. 20	$CHCl_3$:MeOH =1:1	160.3	50	NA
Fr. 21	$CHCl_3$:MeOH =1:1	60.5	NA	NA

[a] Not active at 50 μg/mL (inhibition less than 30%). [b] Inhibition less than 50% but greater than 30%.

Table 2. Non-hydroxylated fatty acids identified by GC-MS.

No.	Retention Time (min)	Molecular Weight	Molecular Formula	Identification	Abbreviation
1	17.28	228	$C_{14}H_{28}O_2$	Myristic acid	C14:0
2	19.58	242	$C_{15}H_{30}O_2$	Pentadecanoic acid	C15:0
3	22.52	256	$C_{16}H_{32}O_2$	Palmitic acid	C16:0
4	24.13	254	$C_{16}H_{30}O_2$	Palmitoleic acid	C16:1/ *cis*-9
5	26.60[a]	252	$C_{16}H_{28}O_2$	7, 10-Hexadecadienoic acid	C16:2/ *trans*-7, 10
6	29.49	282	$C_{18}H_{36}O_2$	Stearic acid	C18:0
7	30.08[a]	250	$C_{16}H_{26}O_2$	7, 10, 13-Hexadecatrienoic acid	C16:3/ *trans*-7, 10, 13
8	31.38	280	$C_{18}H_{34}O_2$	Oleic acid	C18:1/ *cis*-9
9	31.73[a]	280	$C_{18}H_{34}O_2$	13-Octadecenoic acid	C18:1/ *cis*-13
10	34.75	278	$C_{18}H_{32}O_2$	Linoleic acid	C18:2/ *cis*-9, 12
11	37.03	276	$C_{18}H_{30}O_2$	γ-Linolenic acid	C18:3/ *cis*-6, 9, 12
12	38.79	276	$C_{18}H_{30}O_2$	α-Linolenic acid	C18:3/ *cis*-9, 12, 15
13	43.64	308	$C_{20}H_{36}O_2$	11, 14-Eicosadienoic acid	C20:2/ *cis*-11, 14
14	46.16	306	$C_{20}H_{34}O_2$	8, 11, 14-Eicosadienoic acid	C20:3/ *cis*-8, 11, 14
15	47.11	340	$C_{22}H_{44}O_2$	Behenic acid	C22:0
16	48.00	302	$C_{20}H_{32}O_2$	Arachidonic acid	C20:4/ *cis*-5, 8, 11, 14
17	56.24	368	$C_{24}H_{48}O_2$	Lignoceric acid	C24:0

[a]Identified by databases (Wiley and NIST).

Table 3. ^{1}H and ^{13}C NMR data of pheophorbide a (**26**) in $CDCl_3$.

**Pheophorbide a (26)**		
**Position**	**δ_C**	**δ_H**
1	142.1	
2	131.9	
2^1	12.2	3.41 s
3	136.1	
3^1	129	8.0 dd
3^2	122.7	6.19 d
4	136.5	
5	97.5	9.37 s
6	155.6	
7	136.2	
7^1	11.2	3.24 s
8	145.2	
8^1	19.4	3.69 q
8^2	17.5	1.71 t
9	149.8	
10	104.4	9.50 s
11	137.9	
12	129	
12^1	12.2	3.61 s
13	128.9	
13^1	189.7	3.88 s
13^2	53	6.25 s
13^2	64.8	
13^2	169.8	
14	151.0	
15	105.2	
16	161.4	
17	51.2	4.22 brd
17^1	31.2	2.61 m
17^2	29.8	2.31 m
17^3	178.3	
18	50.2	4.46 q
18^1	23.3	1.82 d
19	172.3	
20	93.2	8.56 s

Table 4. ^1H and ^{13}C NMR data of MGDG-1 (**31)** in $CDCl_3$.

Position		δH		δC	
1		4.37 m		62.8	
		4.16 m			
2		5.30 m		70.3	
3		3.86 m		67.9	
		3.75 m			
1'		4.21 m		103.9	
2'		3.65 m		71.1	
3'		3.59 m		73.4	
4'		3.94 m		68.7	
5'		3.48 m		74.6	
6'		3.95 m		61.2	
		3.86 m			
1"	1'''			172.6	173.7
2"	2'''	2.31 br	2.24 m	34.0	34.0
3"	3'''	1.53 m	1.53 m	22.5	24.8
4"	4'''	5.30 m	1.24 m	127.7	29.1
5"	5'''	5.30 m	1.24 m	128.5	29.2
6"	6'''	2.00 m	1.24 m	27.1	29.1
7"	7'''	5.30 m	1.24 m	129.3	29.6
8"	8'''	5.30 m	1.99 m	127.7	27.1
9"	9'''	2.74 m	5.30 m	25.5	130.1
10"	10'''	5.30 m	5.30 m	128.2	127.6
11"	11'''	5.30 m	2.74 m	128.3	25.5
12"	12'''	2.74 m	5.30 m	25.5	127.8
13"	13'''	5.30 m	5.30 m	127.0	128.1
14"	14'''	5.30 m	2.74 m	131.9	25.5
15"	15'''	2.00 m	5.30 m	20.5	126.9
16"	16'''	0.91 t (7.0)	5.30 m	14.2	131.8
	17'''		1.99 m		20.5
	18'''		0.82 m		14.0

Table 5. ^{1}H and ^{13}C NMR data of hydro-pheophorbide-lactone a (**42**) in pyridine-d_6.

Hydro-pheophorbide-lactone a (42)		
Position	δ_C	δ_H
1	141.7	
2	132.4	
2^1	12.5	3.41 s
3	136.4	
3^1	129.8	8.22 dd, 11.5, 17.8
3^2	123.0	6.18 d, 11.5; 6.43 d, 17.8
4	136.6	
5	100.3	9.87 s
6	156.2	
7	137.3	
7^1	11.6	3.27 s
8	146.3	
8^1	20.1	3.80 q, 7.6
8^2	18.2	1.75 t, 7.5
9	150.7	
10	104.8	10.05 s
11	139.1	
12	132.3	
12^1	12.5	4.00 s
13	115.5	
13^1	163.7	
14	136.3	
15	100.3	
15^1	105.1	
15^2	171.0	
15^3	53.7	3.74
16	168.8	
17	54.6	5.22 d, 8.9
17^1	33.2	2.43 m; 3.09 m
17^2	33.8	2.80 m; 3.03 m
17^3	176.2	
18	51.0	4.72 d, 7.3
18^1	23.1	1.81 d, 7.1
19	172.8	
20	95.2	9.06 s

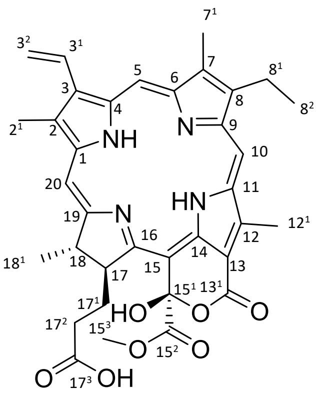

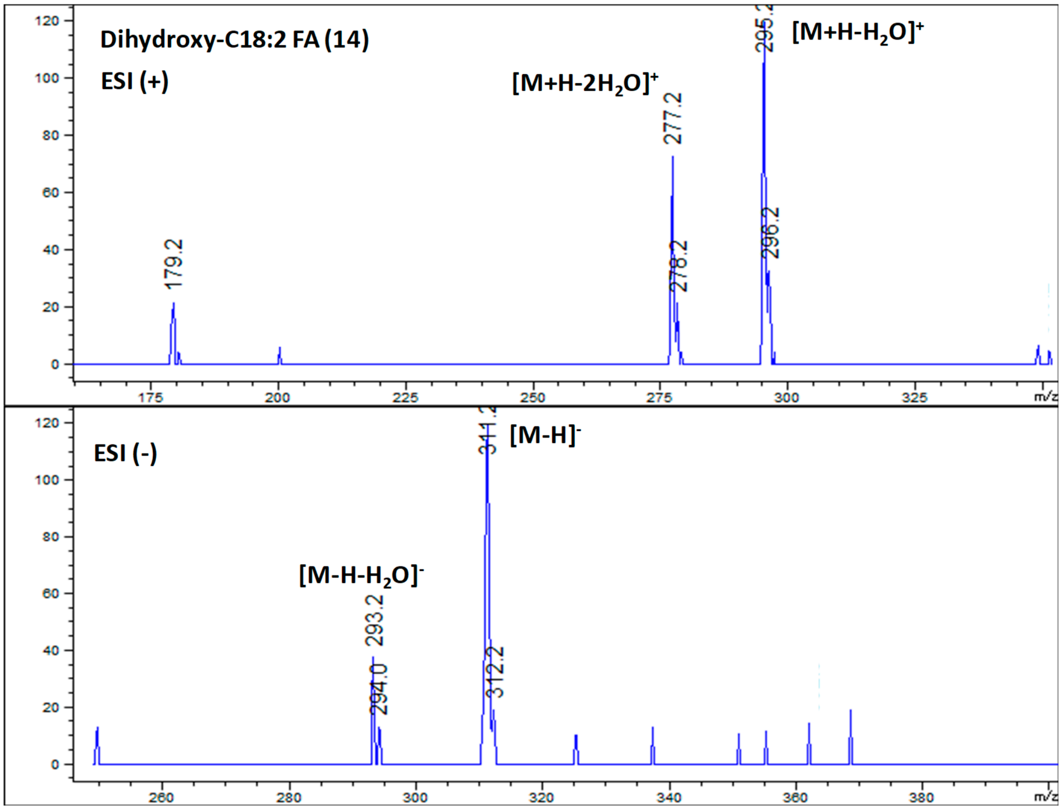

Figure 1. ESI-MS spectra of diydroxylated fatty acid compound **14** obtained in ESI (+) and ESI (-).

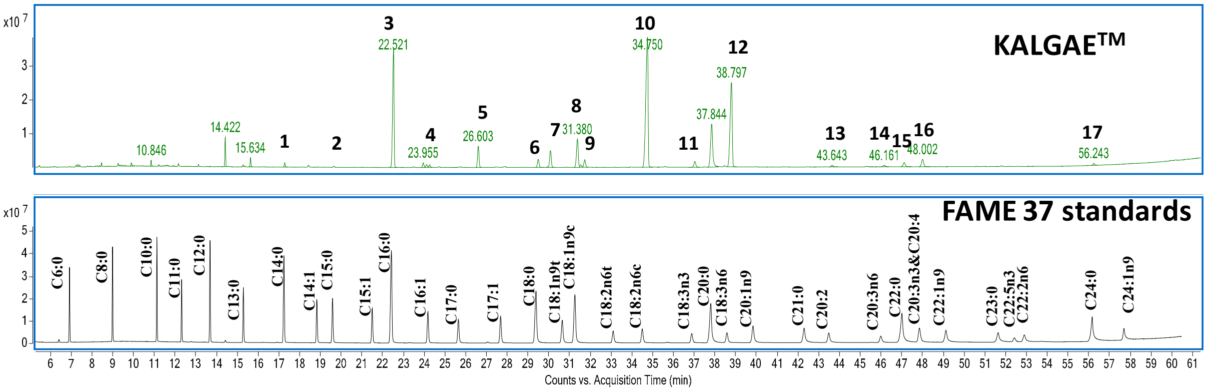

Figure 2. Total ion chromatograms of fatty acid methyl esters of KALGAE™ and FAME standards (C4:0 with a retention of less than 6 min and C22:6n3 with a retention time of greater than 60 min are not shown).

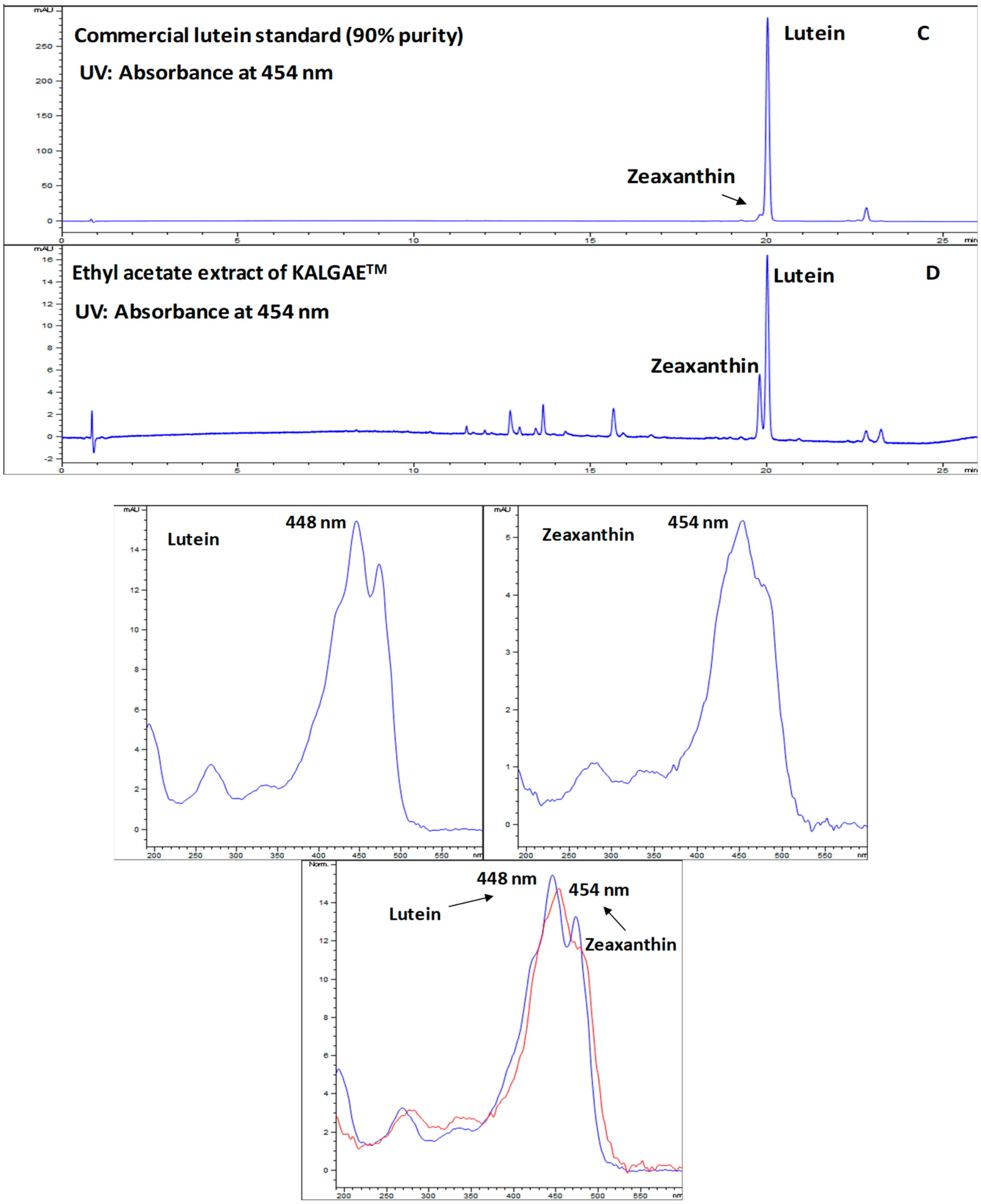

Figure 3. Differentiation of lutein from zeaxanthin by LC-MS-UV.

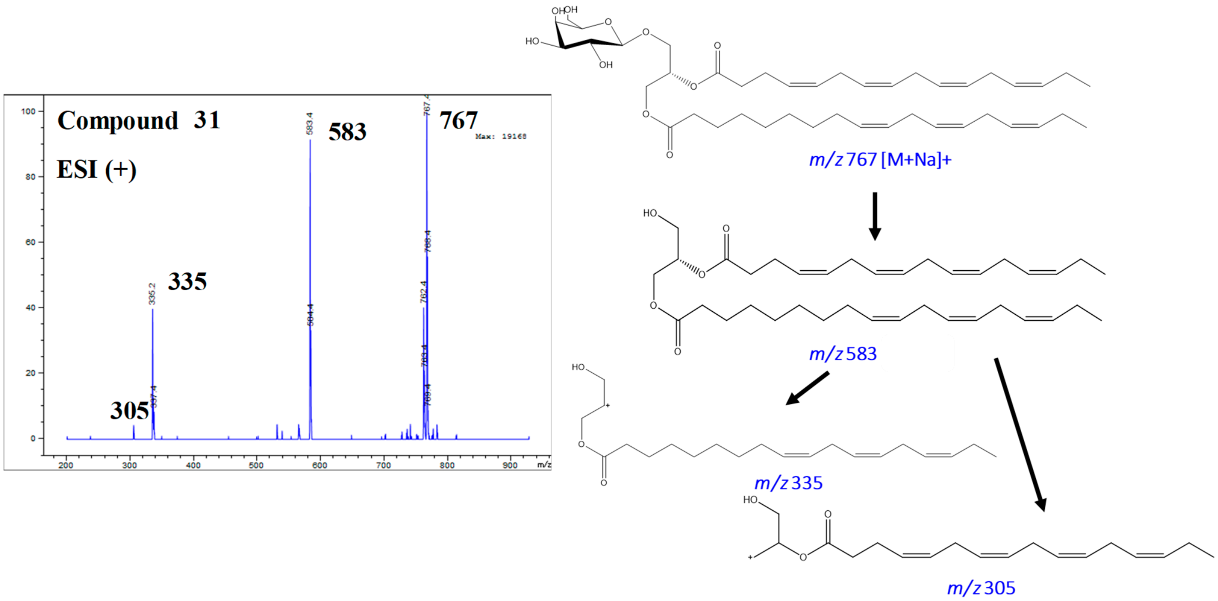

Figure 4. ESI-MS fragmentation patterns of monogalactosyldiacyglycerol compound **31**.

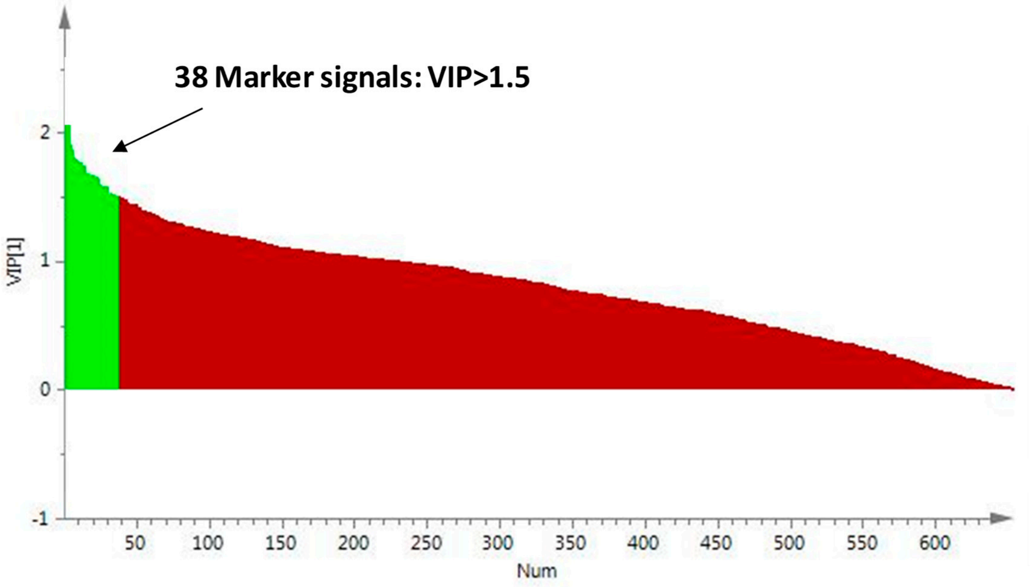

Figure 5. 654 signals sorted in VIP values from high to low.

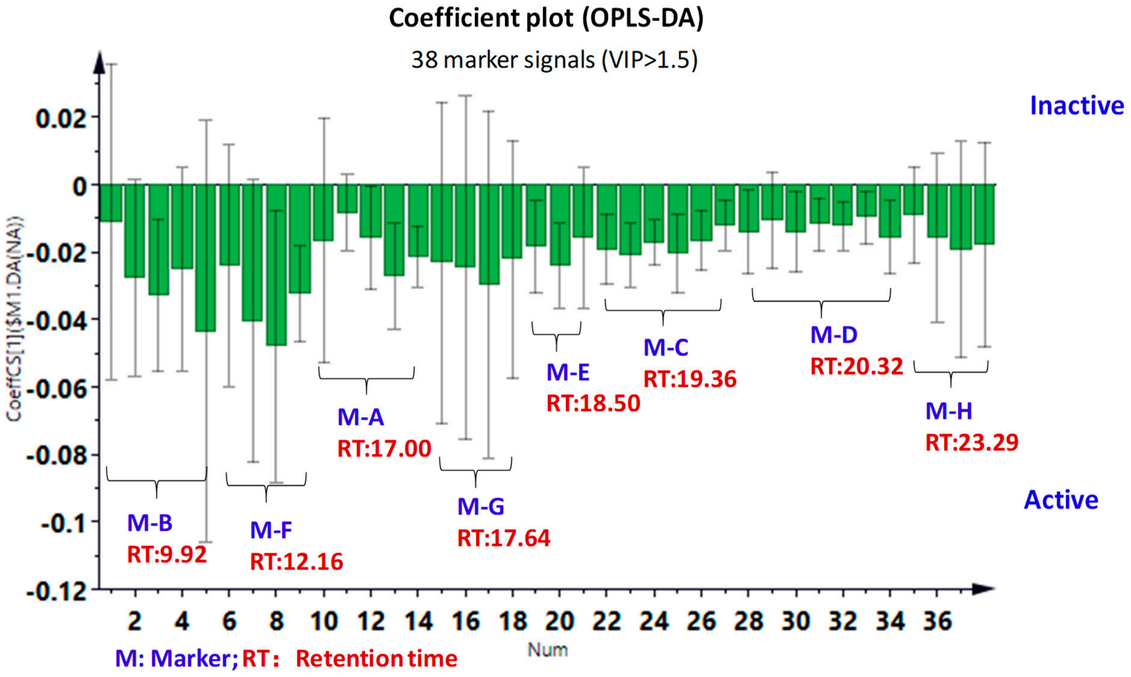

Figure 6. OPLS-DA coefficient plot of chromatographic signals of 21 column fractions generated from the positive ESI-MS: 8 compounds were identified to correspond to 38 marker signals.

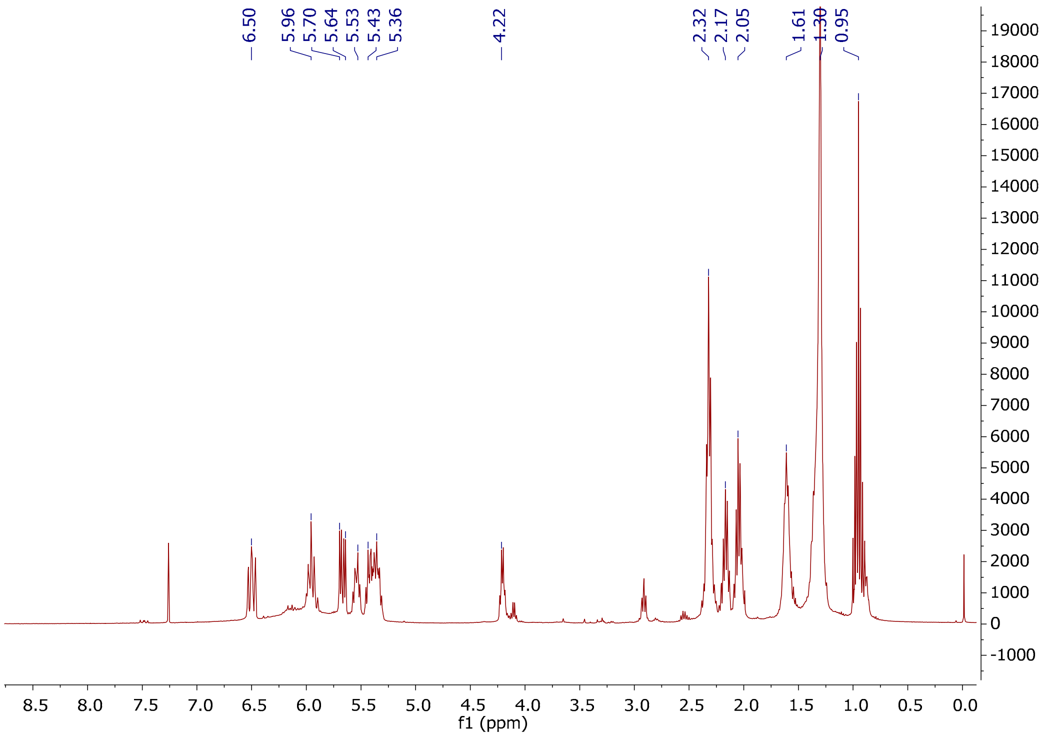

Figure 7. 400 MHz ^{1}H NMR spectrum of (10*E*,12*Z*,15*Z*)-9-hydroxyoctadecadienoic acid (**21**) in $CDCl_3$.

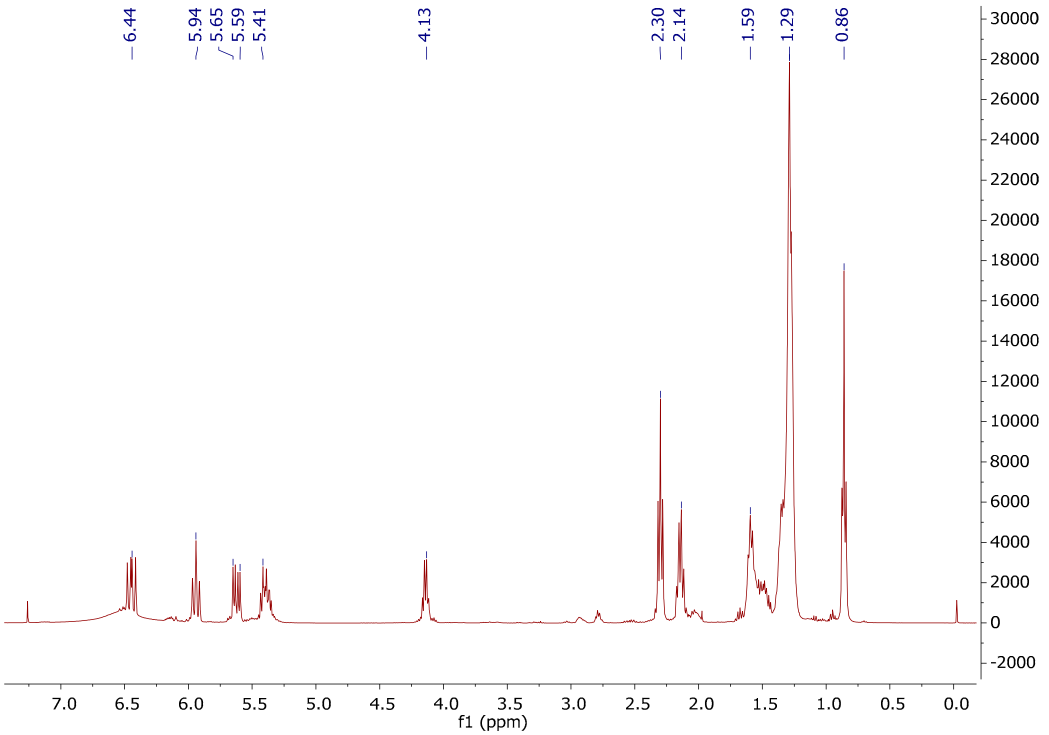

Figure 8. 400 MHz ^1H NMR spectrum of (10*E*,12*Z*)-9-hydroxyoctadecadienoic acid (**22**) in $CDCl_3$.

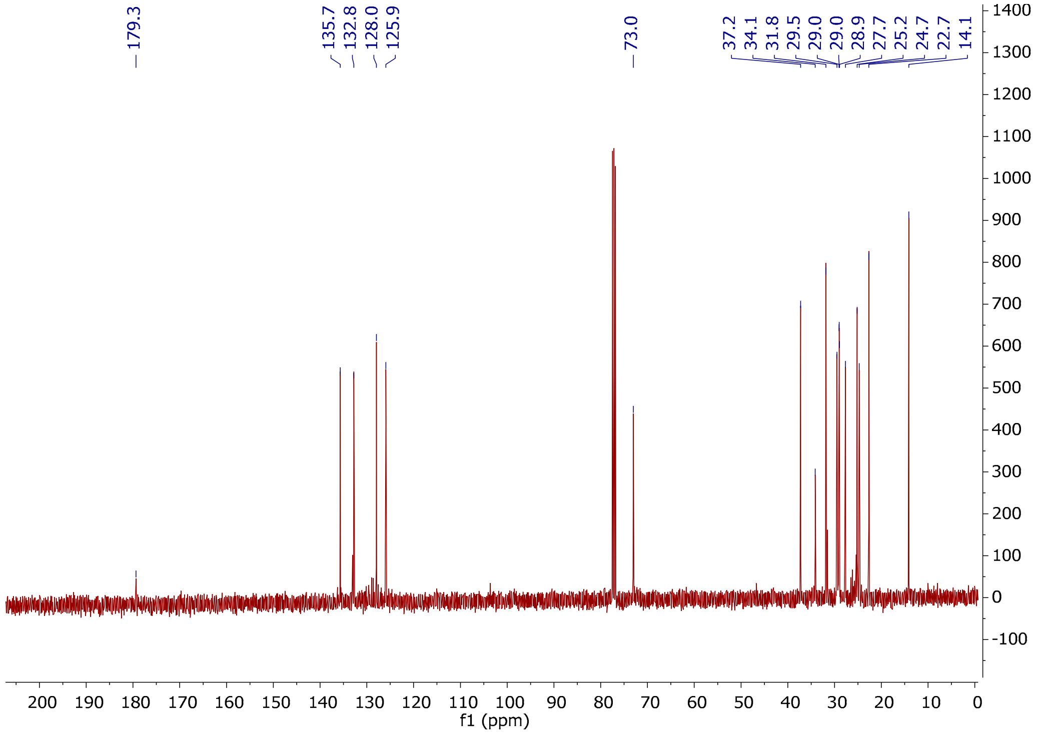

Figure 9. 100 MHz ^{13}C NMR spectrum of (10*E*,12*Z*)-9-hydroxyoctadecadienoic acid (**22**) in $CDCl_3$.

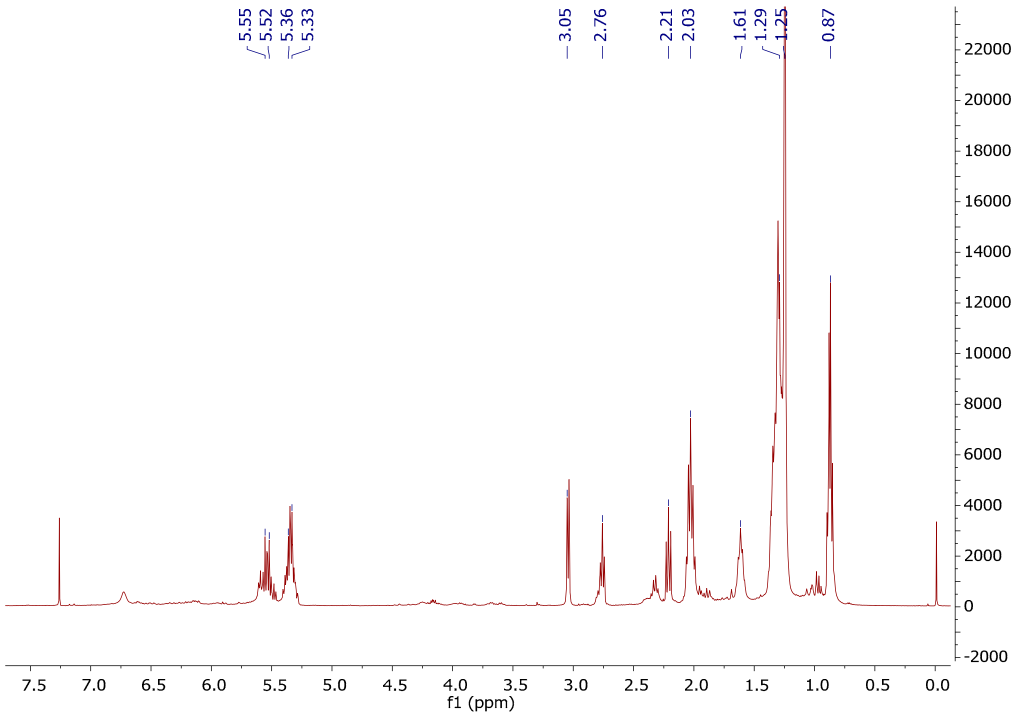

Figure 10. 400 MHz ^{1}H NMR spectrum of (9*Z*,12*Z*)-octadecadienamide (**24**) in $CDCl_3$.

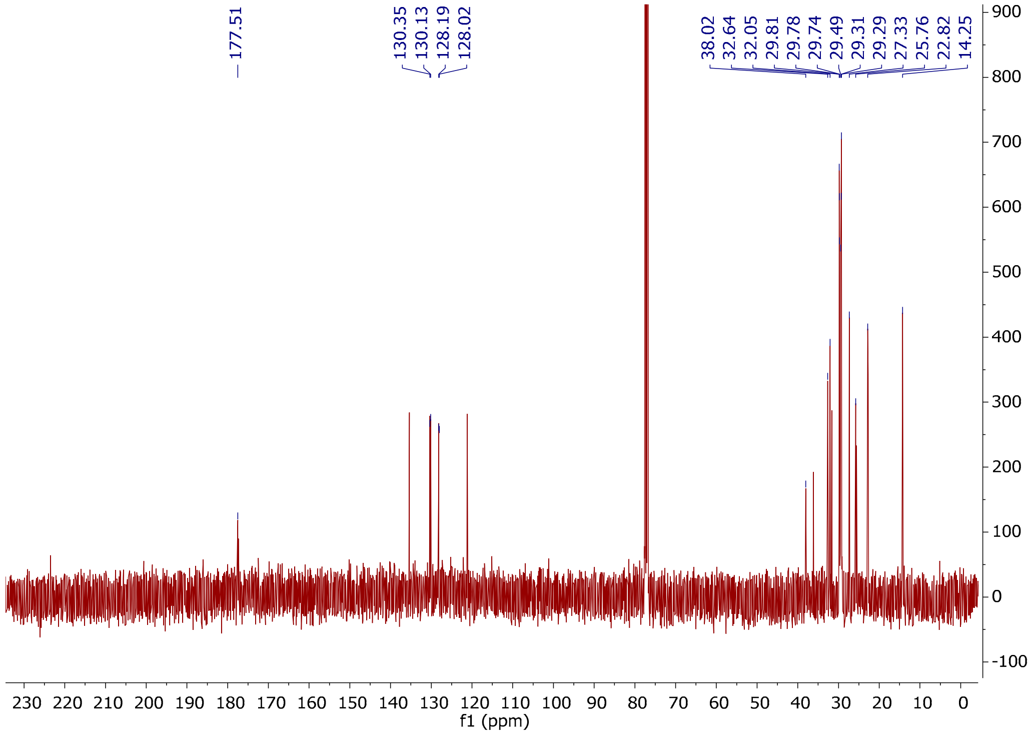

Figure 11. 100 MHz ^{13}C NMR spectrum of (9*Z*,12*Z*)-octadecadienamide (**24**) in $CDCl_3$.

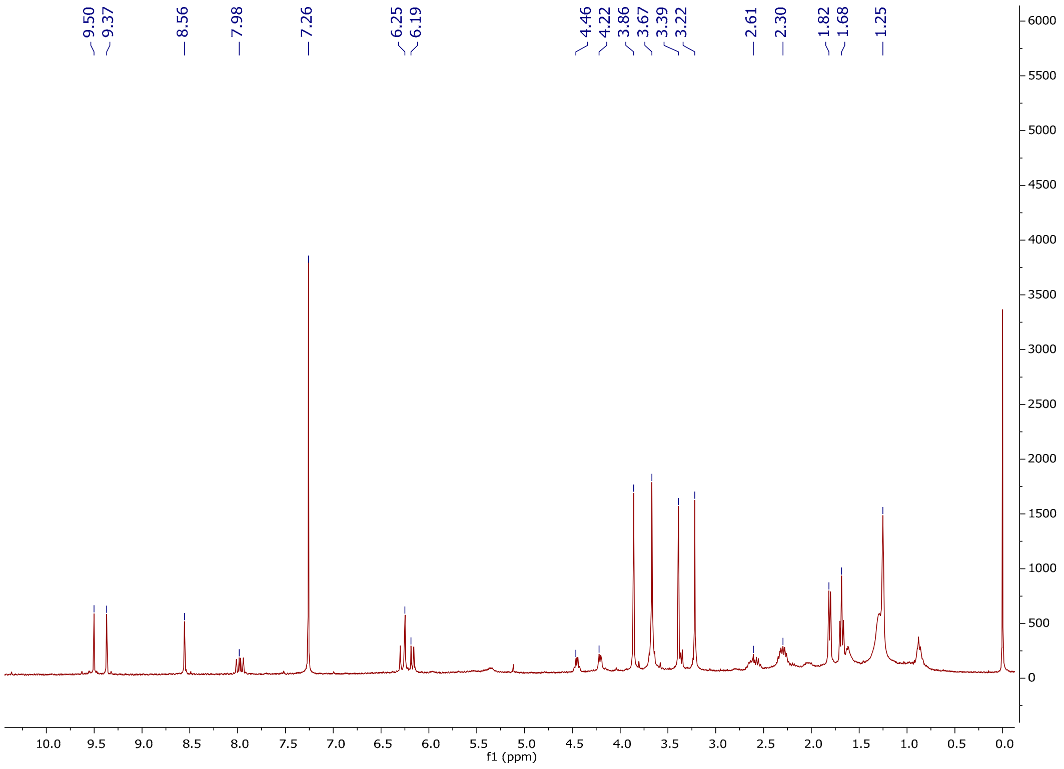

Figure 12. 400 MHz ^{1}H NMR spectrum of pheophorbide a (**26**) in $CDCl_3$.

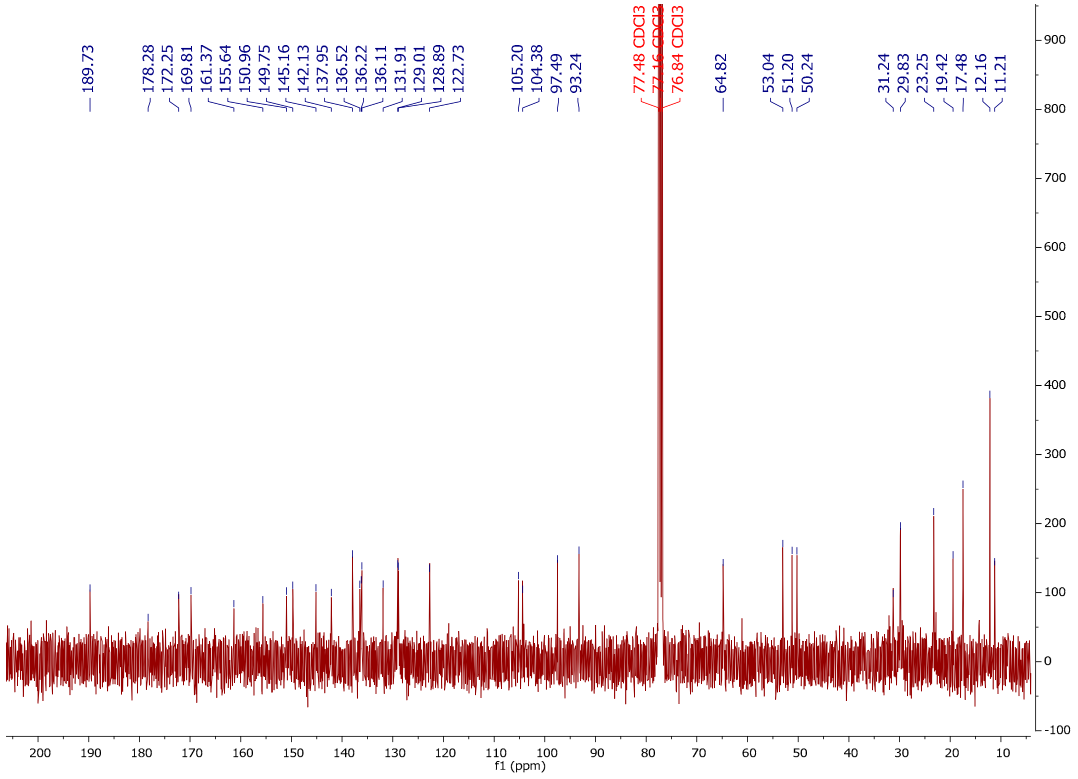

Figure 13. 100 MHz ^{13}C NMR spectrum of pheophorbide a (**26**) in $CDCl_3$.

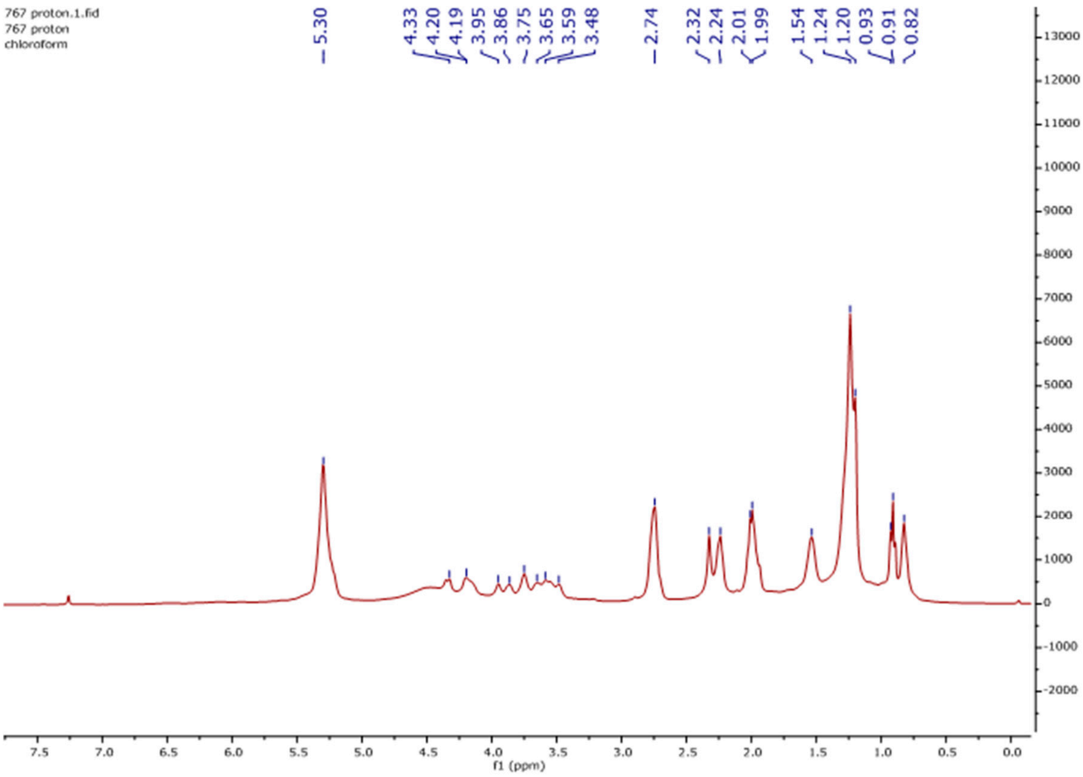

Figure 14. 400 MHz ¹H NMR spectrum of (2*S*)-1-*O*-(9Z,12Z,15Z-octadecatrinoyl)-2-*O*-(4Z,7Z,10Z,13Z-hexadecatetranoyl)-3-*O*-*β*-D-galacto pyranosylglycerol (**31**) in $CDCl_3$.

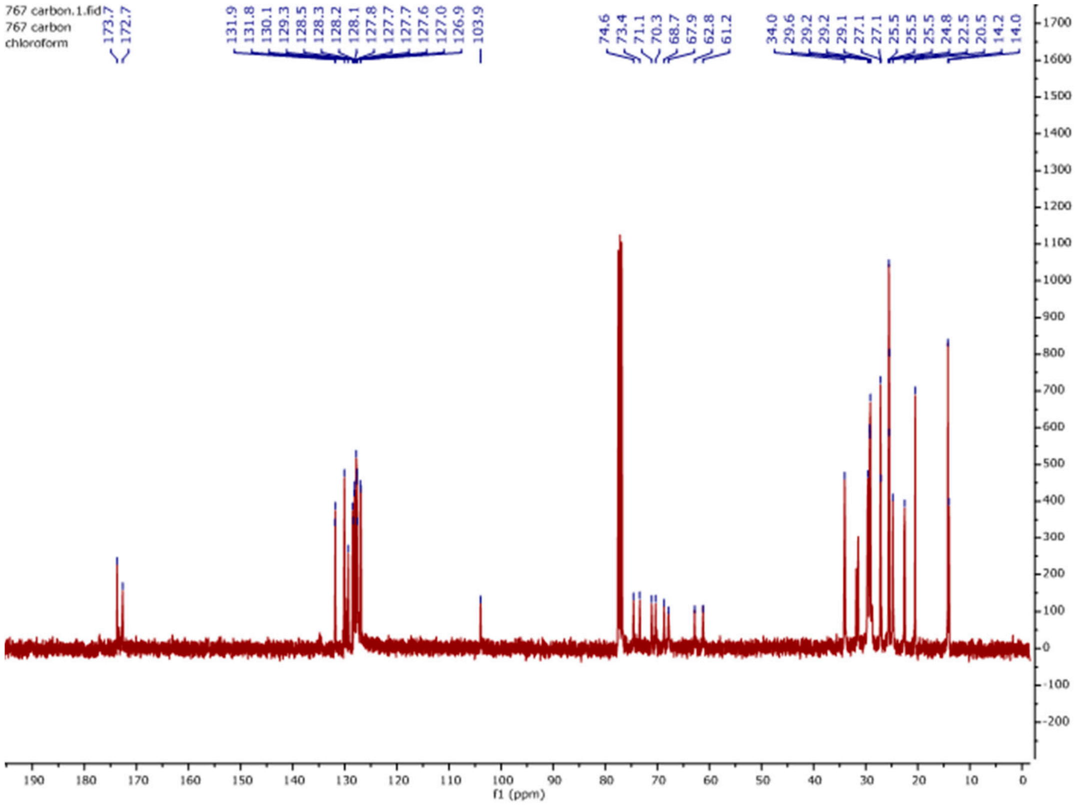

Figure 15. 100 MHz ¹³C NMR Spectrum of (2*S*)-1-*O*-(9*Z*,12*Z*,15*Z*-octadecatrinoyl)- 2-*O*-(4Z,7Z,10Z, 13*Z*-hexadecatetranoyl)-3-*O*-*β*-D-galactopyranosylglycerol (**31**) in $CDCl_3$.

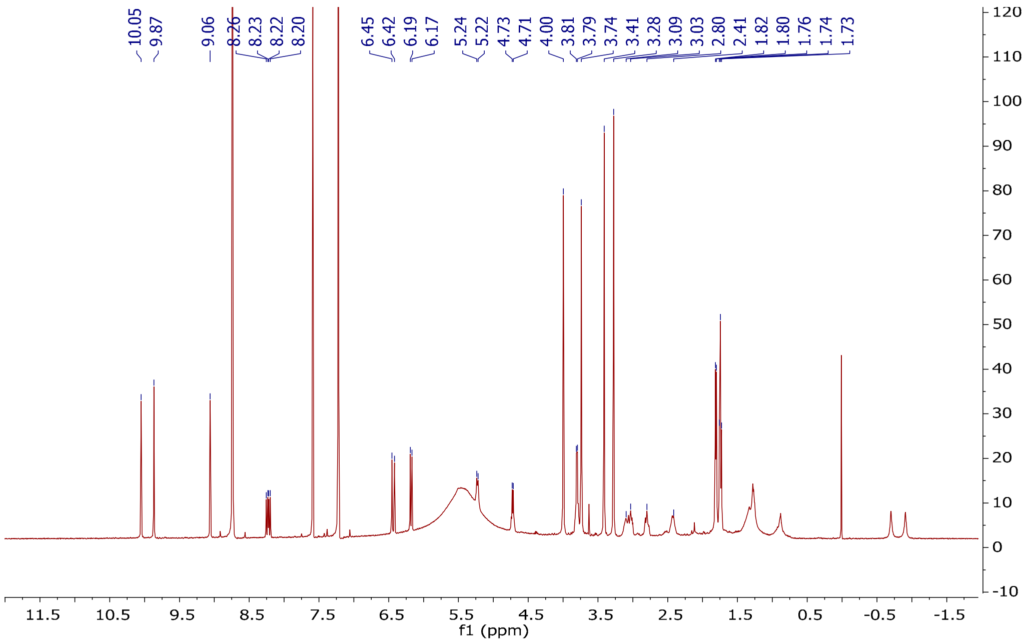

Figure 16. 500 MHz ^{1}H NMR spectrum of hydro-pheophorbide-lactone a (**42**) in pyridine-d_6.

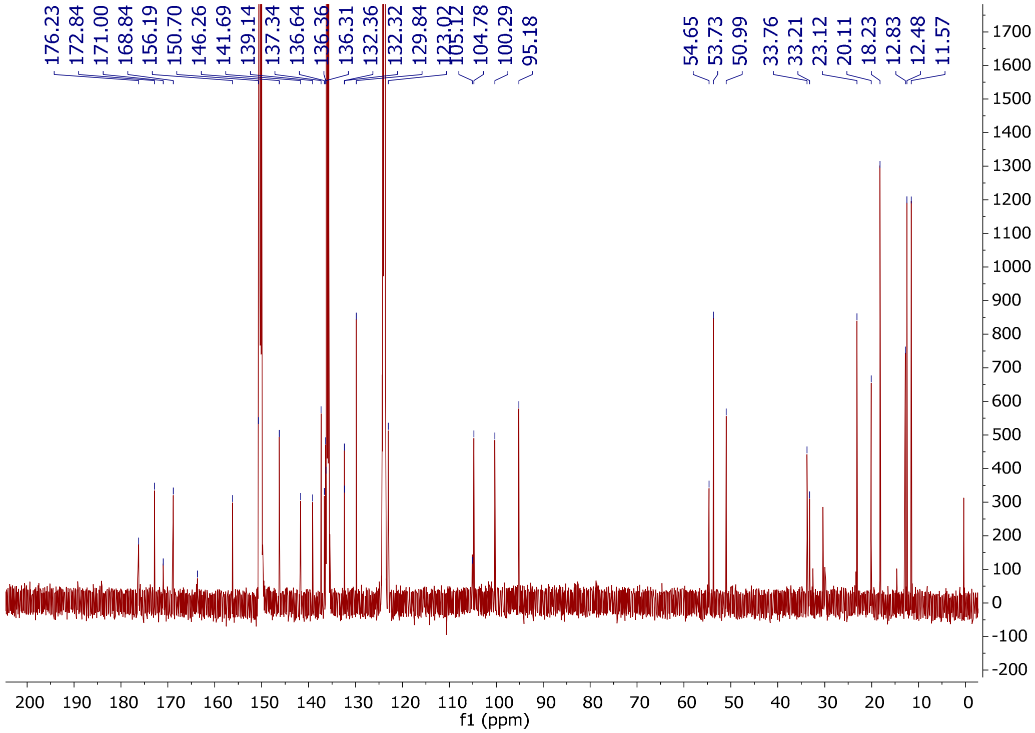

Figure 17. 125 MHz ^{13}C NMR spectrum of hydro-pheophorbide-lactone a (**42**) in pyridine-d_6.

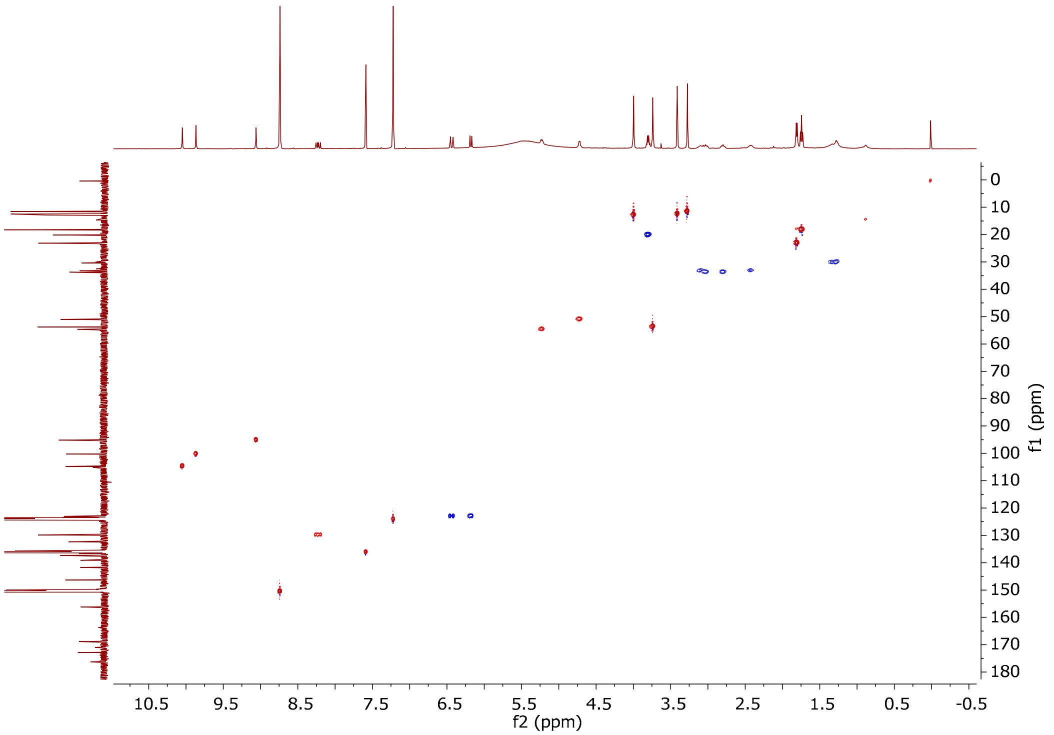

Figure 18. 500 MHz HSQC NMR spectrum of hydro-pheophorbide-lactone a (**42**) in pyridine-d_6.

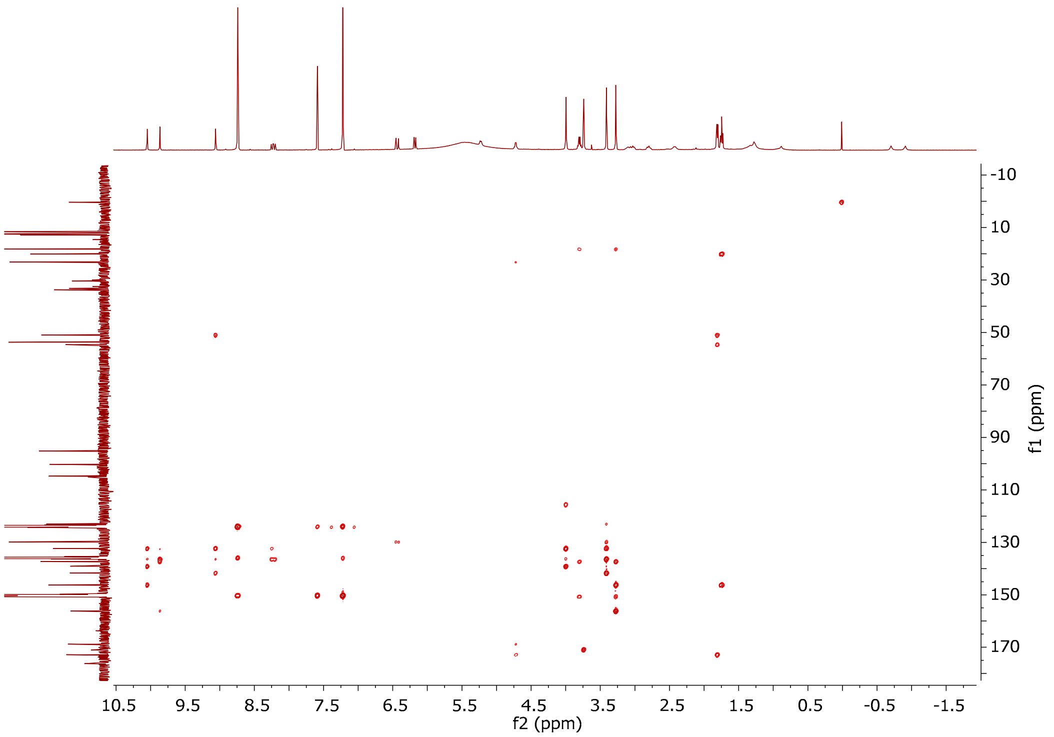

Figure 19. 500 MHz HMBC NMR spectrum of hydro-pheophorbide-lactone a (**42**) in pyridine-d_6.

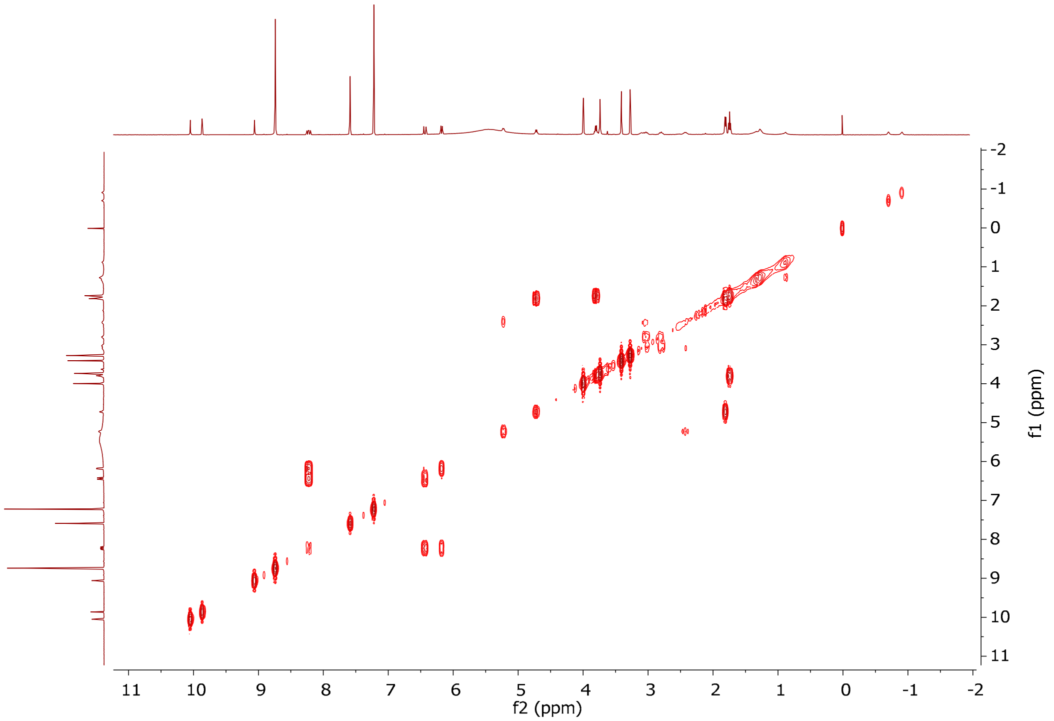

Figure 20. 500 MHz COSY NMR spectrum of hydro-pheophorbide-lactone a (**42**) in pyridine-*d*6.

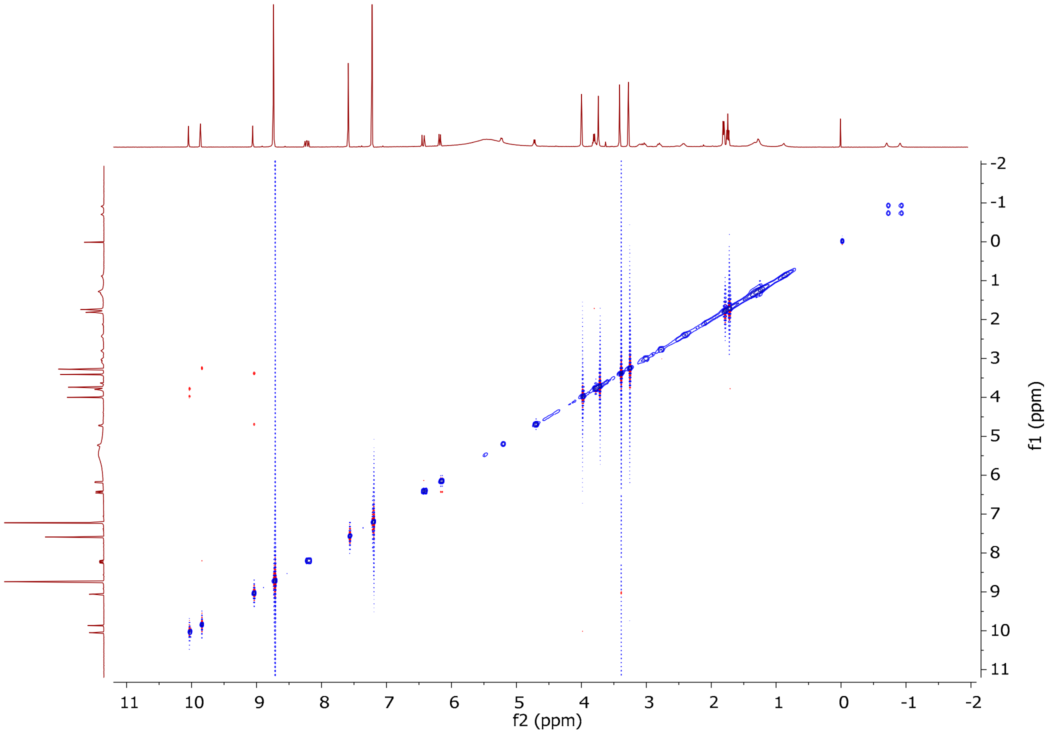

Figure 21. 500 MHz ROESY NMR spectrum of hydro-pheophorbide-lactone a (**42**) in pyridine-*d*6.
